# Comment on Fan et al. Efficacy of Ingesting an Oral Rehydration Solution after Exercise on Fluid Balance and Endurance Performance. *Nutrients* 2020, *12*, 3826

**DOI:** 10.3390/nu13093214

**Published:** 2021-09-16

**Authors:** Charles Dumke

**Affiliations:** School of Integrative Physiology and Athletic Training, University of Montana, 32 Campus Drive, 206 McGill Hall, Missoula, MT 59812, USA; charles.dumke@umontana.edu; Tel.: +1-406-243-6176; Fax: +1-406-243-6252

Our lab read with interest the recent article published in *Nutrients* comparing different drink composition on fluid balance [1], as we have performed similar research [2]. It appears several aspects of this study deserve comment.

Noticeably, the one statistically significant finding of fluid retention is misleadingly presented. In the text, values are presented as 30%, 10% and −4% for DD, SD and WA, respectively. Yet, in Figure 1b, these numbers are presented as 42%, 25% and 16% for DD, SD and WA, respectively. When one completes the math from the means, it appears the numbers presented in the text are correct. This became obvious when the mean “-“ in.

Figure 1b does not appear to represent the mean of the individual values. We also would have appreciated the level of dehydration that occurred during exercise, as this is how their fluid recovery scheme was determined. From the data presented (1.5 mL/kg/15 min during exercise and 150% post-exercise), the participants received ~400 mL during the 75 min cycling bout, became ~1.5% dehydrated, leaving ~1400 mL as a bolus post-exercise. This would certainly present a challenge to retention, which is supported by the resulting urine output of >1.5 L.

Although this brings into question the data presentation, we do not doubt the modest, yet significant, effect of DD on rehydration as this confirms previous literature. However, it is also telling that this was the only outcome that reached statistical significance. Not mentioned, but understood, is the powerful effect of the exercise induced antidiuretic hormones on fluid retention. Indeed, it was only during the thermoneutral 5 h of recovery, once these hormones were attenuated, that a small difference in fluid retention was captured. In our previous study, we did not include a long post-exercise recovery as our interest was fluid retention during exercise, which had not been previously compared between SD and DD. Oral rehydration solutions were originally formulated by the WHO to promote rehydration following diarrhea in the absence of intravenous saline [3]. Although it may be effective in this capacity, one cannot extrapolate its effectiveness to during exercise in the heat, for which it is often marketed. Others have also suggested that ideal fluid constituents differ depending on the situation and method of dehydration [4].

Practically, it is important to note that only under the conditions of thermoneutral rest for 5 hours, following a large bolus of fluid, in the absence of ingestion of any other nutrition, were small differences in fluid retention found. For workers or athletes in the field, this is not a realistic scenario and, thus, lacks external validity.
